# The genome profiling method can be applied for species identification of biological materials collected at crime scenes

**DOI:** 10.1186/s12863-019-0753-9

**Published:** 2019-06-10

**Authors:** Takako Kinebuchi, Nozomi Idota, Hajime Tsuboi, Marin Takaso, Risa Bando, Hiroshi Ikegaya

**Affiliations:** Department of Forensic Medicine, Graduate School of Medical Science, Kyoto Prefectural University of Medicine, 465 Kajiicho, Kamigyo, Kyoto, 602-8566 Japan

**Keywords:** Forensic science, Genome profiling, Species identification, Detection, Classification, Random primers

## Abstract

**Background:**

Various biological materials unrelated to humans are found at crime scenes and it is often important to elucidate the origin of these materials. A genetic locus common to several species is conventionally PCR-amplified with universal primers to identify species. However, not all species can be identified using a single locus.

In this study, DNA from 13 commonly handled taxa was analyzed to identify species by a genome profiling (GP) method, which involves random PCR and temperature gradient gel electrophoresis.

**Results:**

In a clustering analysis, we successfully obtained a single cluster for each species.

**Conclusion:**

The GP method is cost-effective and does not require advanced techniques and knowledge in molecular biology. The random sampling of the whole genome using multiple primers provides substantial genomic information. Therefore, the method is effective for classifying a wide range of species, including animals, plants, and insects, and is useful for crime scene investigations.

**Electronic supplementary material:**

The online version of this article (10.1186/s12863-019-0753-9) contains supplementary material, which is available to authorized users.

## Background

Several methods to detect human-specific DNA have been developed to identify victims or suspects, using biological materials such as blood stains, hair, small tissue particles, and body fluids found at crime scenes [1–3]. It is important to identify materials derived from humans; however, for materials that are not of human origin, it is often still important to determine the source. For instance, this can be important in cases where feces are placed in front of a house, when hair or bone fragments are mixed in a food manufacturing factory, or when wild animals cause agricultural damage. Species identification from biological materials found at a crime scene can inform subsequent police investigations.

For non-human biological materials, such as meat pieces, blood, hair, and bone fragments, the amplification of a species-specific locus by PCR is commonly performed [4–8]. However, only expected creatures can be investigated using this approach. There are many methods for amplifying sequences common to multiple species using universal primers, such as the amplification of mitochondrial rDNA [9, 10], cytochrome b [11, 12], cytochrome oxidase I [13, 14], myoglobin [15], or the D-loop region [16–20] and subsequent identification using the Basic Local Alignment Search Tool. Even if sequences for many species are registered in the database, the location of the sequence varies. Accordingly, there is a limit to species identification based on a single locus. In addition, even if samples belong to the same species, there may be individual differences in DNA sequences. To address these issues, analyses of additional loci and molecular phylogenetic approaches are needed. However, these methods are relatively expensive and require expertise and equipment.

In this study, we focused on the genome profiling (GP) method. The GP method was developed by Nishigaki et al. in the bioindustry field in 1971 [21]. In the GP method, DNA is PCR-amplified using random primers (random PCR) and temperature gradient gel electrophoresis (TGGE) is performed. Depending on the number of amplified fragments and the melting temperatures of the double-stranded DNA, species identification dots (*spiddos*) are obtained on the temperature gradient gel. These *spiddos* are corrected using internal standards. Then, the *spiddos* pattern is compared between samples and references and a pattern similarity score (PaSS) is obtained. Using this PaSS, a cluster analysis is performed to identify species.

This method corresponds to random sampling in statistics. It is possible to analyze information for the entire genome at a very low cost in a short period of time, without requiring any special knowledge or techniques. Using this GP method, we have identified human body fluid [22, 23] and viral species [24] and differentiated between humans and other mammalian species [25].

The GP method is highly sensitive and accordingly it can potentially be used like the Ames test for mutagen analyses [26]. Additionally, the potential for personal identification in humans has also been reported [27]. In this study, we selected a wide range of target organisms, including common fish, birds, and various mammals, and examined whether it is possible to identify these organisms using the GP method.

## Results

Four or more *spiddos* were obtained for all samples, regardless of species (Table 1). The average number of *spiddos* obtained from each sample was 11.7 ± 3.1 (range 5–21) for SP-1, 12.7 ± 3.8 (range 5–22) for SP-2, and 7.0 ± 3.0 (range 4–16) for SP-3.

**Table 1:** Number of *spiddos* in various animal species, obtained using three different random primers

Species	SP-1	SP-2	SP-3	Total
Cattle (*n* = 4)	9.0 ± 2.0	12.3 ± 3.6	6.5 ± 1.0	27.8 ± 4.6
Pig (*n* = 4)	10.5 ± 1.3	16.0 ± 1.8	6.5 ± 2.1	33.0 ± 4.2
Sheep (*n* = 4)	11.3 ± 1.5	9.75 ± 3.4	5.8 ± 1.0	26.8 ± 4.3
Chicken (*n* = 4)	14.0 ± 4.9	13.0 ± 1.4	9.0 ± 1.2	36.0 ± 5.6
Greater amberjack (*n* = 4)	14.8 ± 3.6	19.0 ± 2.1	6.3 ± 1.9	40.0 ± 2.9
Bigeye tuna (*n* = 4)	14.8 ± 2.5	12.5 ± 1.9	5.3 ± 1.0	32.5 ± 2.4
Silver salmon (*n* = 4)	14.8 ± 2.2	14.8 ± 1.3	6.3 ± 1.3	35.8 ± 3.3
Horse mackerel (*n* = 4)	10.8 ± 0.5	14.8 ± 1.5	5.3 ± 1.9	30.8 ± 1.9
Halibut (*n* = 4)	11.8 ± 2.9	13.0 ± 3.3	5.3 ± 1.0	30.0 ± 4.4
Dog (*n* = 3)	11.0 ± 1.4	12.3 ± 1.7	9.8 ± 2.1	33.0 ± 4.1
Cat (*n* = 3)	10.5 ± 1.3	13.3 ± 3.3	13.0 ± 2.5	36.8 ± 5.5
Rat (*n* = 4)	5. 3 ± 1.0	4,5 ± 1.0	6.5 ± 1.3	16.3 ± 1.5
Human (*n* = 3)	13.3 ± 2.1	11.3 ± 2.5	6.3 ± 1.5	31.0 ± 2.6
Total	11.7 ± 3.1	12.7 ± 3.8	7.0 ± 3.0	

Numbers are shown as averages ± S.D

*n:* number

The average numbers of *spiddos* obtained using SP-1 to − 3 were 27.8 for cattle, 33.0 for pigs, 26.8 for sheep, 36.0 for chickens, 40 for greater amberjack, 32.5 for bigeye tuna, 35.8 for silver salmon, 30.8 for Japanese horse mackerel, 30.0 for Japanese halibut, 33.0 for dog, 36.8 for cat, 16.3 for rat, and 31.0 for human.

**Fig. 1:**
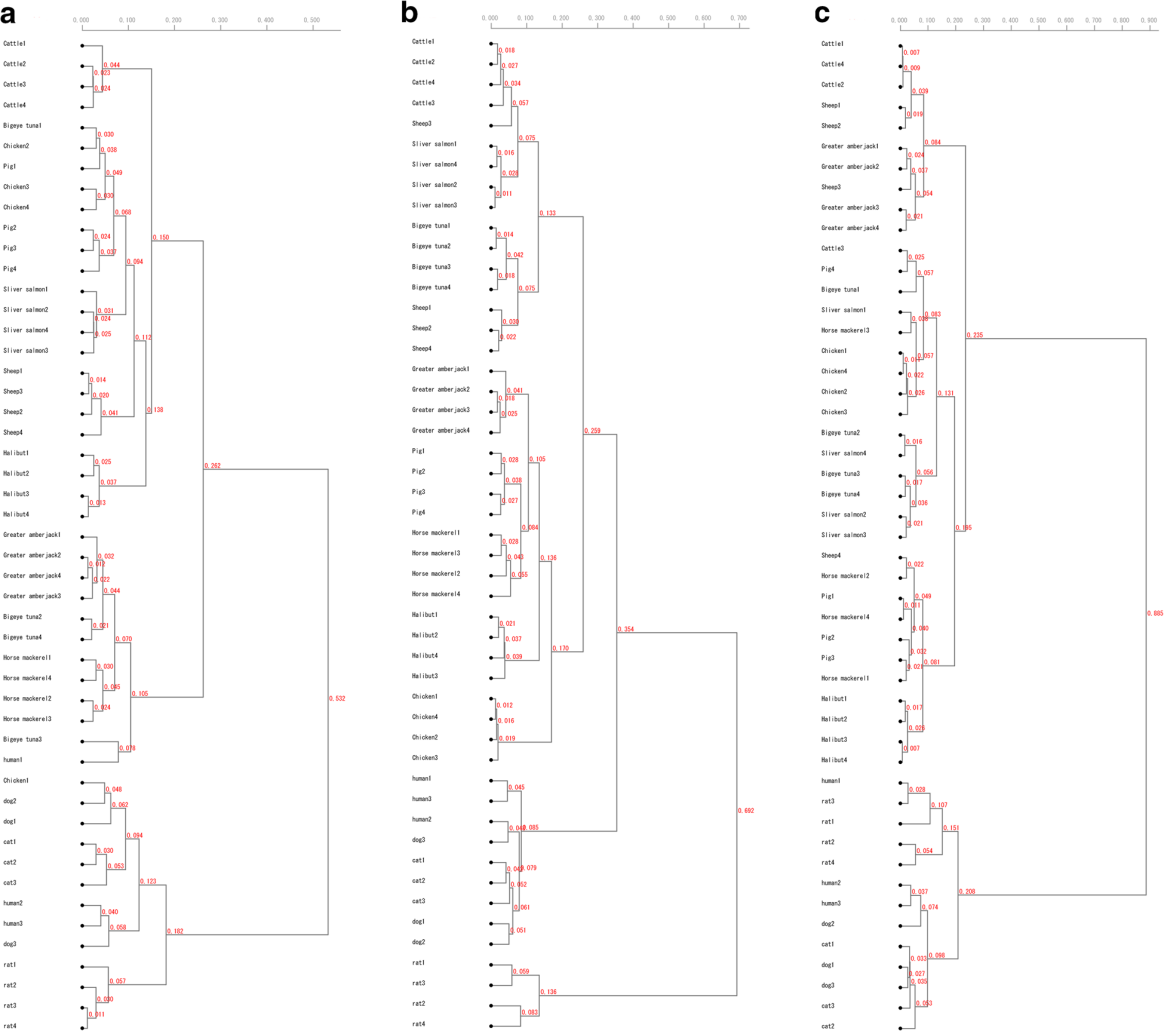
Cluster analysis of 12 animal species and human. Analysis was based on PaSS values calculated from the GP method using the random primer **a** SP-1, **b** SP-2, and **c** SP − 3

Phylogenetic trees were generated using PaSS values for each primer. Using the SP-1 primer, the same species were classified into a single cluster for cattle, sheep, Japanese halibut, greater amberjack, Japanese horse mackerel, and rat. However, mixed clusters were obtained for other species (Fig. 1a). Using the SP-2 primer, the same species were classified into a single cluster for cattle, pig, chicken, greater amberjack, bigeye tuna, silver salmon, Japanese horse mackerel, Japanese halibut, rat, and human. However, mixed clusters were obtained for sheep (Fig. 1b). Using the SP-3 primer, the same species were classified into a single cluster only for chicken and Japanese halibut, and the other species formed mixed clusters (Fig. 1c). Cluster formation was not related to the number of *spiddos.*

**Fig. 2:**
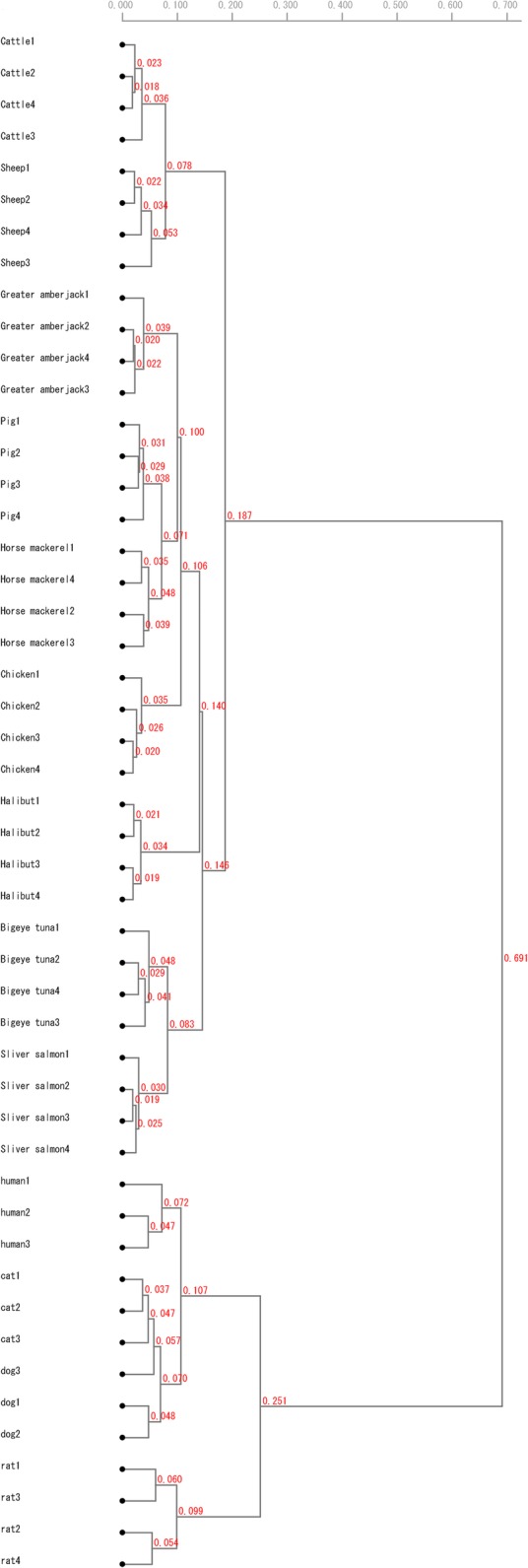
Cluster analysis of 12 animal species and human. Analysis was based on PaSS values calculated by the GP method using the random primers SP-1, − 2, and – 3. Different samples from the same individuals were classified into the same clade

When a cluster analysis was performed based on average PaSS values for SP-1, SP-2, and SP-3, samples from the same species were classified into the same cluster for all species (Fig. 2).

## Discussion

In the field of forensic medicine, DNA typing by short tandem repeat (STR) analysis is generally accepted for the personal identification of biological materials such as blood, semen, and saliva stains collected at a crime scene [28, 29]. However, it is often presumed that blood stains are human in origin. First, it is important to confirm whether the material is derived from humans. For non-human samples, a universal method for species identification is needed.

Though there is a report that simple sequence repeat (SSR) is very effective for determining the differences among species [30], many reports using a genetic approach, including STR or other methods, determined the differences in the same species. The GP method can be used to detect all species, including humans, animals [25], insects, plants [31], bacteria [32], and fungi [33], following a single protocol. Using the GP method, results can be obtained in a few hours, including random PCR and 10 min of TGGE. The cost is only a few dollars per sample.

For the GP method, the accuracy of species determination depends on the number of *spiddos* obtained by random PCR. A *spiddos* shows not only the cleaved temperature of the double-stranded DNA fragment amplified by random PCR, but also specific DNA sequences. Therefore, analyzing *spiddos* is the same as performing random sampling for large-scale genome information. For this reason, primers that can amplify a larger number of *spiddos* are useful to maximize the genome information that is obtained.

However, there is a limit to the number of *spiddos* that can be obtained using a single primer. Accordingly, the number of *spiddos* can be increased by increasing the number of random primers, resulting in a more accurate cluster analysis [33]. In our study, we were able to classify species more accurately using the average value of the results obtained using three primers (Fig. 2) than using each of the three primers separately (Fig. 1a, b, and c). By using multiple random primers, even closely related species, such as the cattle and sheep groups or the greater amberjack and horse mackerel groups, can be classified correctly. Our results indicate that species identification by the GP method using multiple random primers is very accurate.

Classification based on a very small part of the whole genome is sometimes difficult for closely related species. Moreover, the reliability of the results may be insufficient when the whole genome is not analyzed. The GP method, in which the whole genome is analyzed by random sampling, addresses these limitations and is sufficient for species classification. However, the GP method has various limitations. First, it is difficult to apply to mixed samples. Although mixed samples were not examined in this report, both genomes are expected to be randomly amplified, and it may be difficult to determine their species. In previous studies of virus detection using body fluids, we found species-specific *spiddos* [23, 24]. There may be specific *spiddos* in various species. Further studies are required to evaluate this species specificity. A second problem is related to the reference samples. Although there are international databases for DNA sequences, a database of TGGE images does not exist. Therefore, it is necessary to prepare DNA for the suspected species once to obtain images for comparison.

## Conclusion

Despite the issues described above, the GP method can save time, labor, and costs, and although it is a random sampling approach, it can be used to obtain whole genome information. Therefore, the GP method is also an effective approach for classifying species and can be used for criminal investigations.

## Methods

### Samples

Human and 12 species that are often found in the house or household kitchens were used in this study, i.e., cattle *(Bos taurus*), pig (*Sus scrofa domesticus*), sheep (*Ovis aries*), chicken (*Gallus Gallus domesticus*), greater amberjack (*Seriola dumerili*), bigeye tuna (*Thunnus obesus*), silver salmon (*Oncorhynchus kisutch*), Japanese horse mackerel (*Trachurus japonicus*), Japanese halibut (*Paralichthys olivaceus*), dog (*Canis lupus familiaris*), cat (*Felis catus*), and rat (*Rattus norvegicus*). The meats of cattle, pig, sheep, chicken, greater amberjack, bigeye tuna, silver salmon, Japanese horse mackerel, and Japanese halibut were purchased at a grocery store in the city market. Samples were collected by scrubbing the surface of each raw meat with a cotton swab. The dog, cat, and rat samples were obtained at pet shops by scrubbing the buccal mucosa of each species with a cotton swab. Human samples were obtained from healthy adult volunteers who provided written informed consent. The buccal mucosa of each volunteer was scrubbed with a cotton swab. A total of 49 samples (3–4 samples for each animal species and 3 samples for human) were collected.

Cotton swabs were digested with Proteinase K overnight at 56 °C. DNA was extracted using QIAamp® DNA Mini Kits (Qiagen, Tokyo, Japan). The DNA concentration was adjusted to 5 ng/μL.

### Random PCR

Three random primers, SP-1 (pfm12) (5′-AGAACGCGCCTG-3′), SP-2 (pfm19) (5′-CAGGGCGCGCGTAC-3′), and SP-3 (hunt) (5′-TGCTGCTGCTGC-3′) were used [34]. PCR amplification was performed using a 50-μL reaction solution containing 4.0 μL of dNTP (2.5 mM each), 5.0 μL of Buffer, 3.5 μL of Ex Taq Polymerase (Takara Bio Inc., Shiga, Japan), 10 mM each primer, and 1.0 μL of extracted DNA. PCR was performed using the PC-320 Thermal Cycler (ASTEC, Fukuoka, Japan) as follows: 30 cycles of 94 °C for 30 s, 26 °C for 1 min, and 47 °C for 1 min, and a final extension at 47 °C for 5 min.

### Internal standards

Two types of reference DNA were prepared as TGGE internal standards [35]. For reference 1 (Ref1), PCR amplification was performed using a 50-μL reaction solution containing 2.0 μL of M13 phage DNA (TaKaRa Bio, Inc.), 3.0 μL each of 10 μM MA1 (5′-TGCTACGTCTCTTCCGATGCTGTCTTTC-3′) and MA2 (5′-CCTTGAATTCTATCGGTTTATCA-3′), 4.0 μL of dNTP (2.5 mM each), 5.0 μL of 10× Buffer, 0.15 μL of Ex Taq Polymerase (Takara Bio Inc.), 10 mM primers, and 1.0 μL of extracted DNA. PCR conditions were 30 cycles of 94 °C for 30 s, 63 °C for 1 min, and 72 °C for 30 s, and a final extension at 72 °C for 5 min.

For reference 2 (Ref2), PCR amplification was performed using a 50-μL reaction solution containing 2.0 μL of M13 phage DNA (TaKaRa Bio, Inc.), 3.5 μL each of 10 μM Ref6F (5′-GCCGGCATCACCGGCGCCACAGGTGCGGTTG-3′) and Ref6R (5′-TAGCGAGGTGCCGCCGGCTTCCATTCAGGTC-3′), 4.0 μL of dNTP (2.5 mM each), 5.0 μL of 10× Buffer, 0.25 μL of Ex Taq Polymerase (Takara Bio Inc.), 10 mM primers, and 1.0 μL of extracted DNA. PCR conditions were 30 cycles of 94 °C for 15 s, 44 °C for 30 s, and 72 °C for 1 min, and a final extension at 72 °C for 30 s.

### Temperature gradient gel electrophoresis (TGGE)

A total of 1.0 μL of reference DNA solution was obtained by mixing the reaction solutions of Ref1 and Ref2 at a ratio of 1:1 and 1.0 μL of the PCR solution, followed by electrophoresis. The mixed sample was applied to a 6% polyacrylamide gel and electrophoresed at 100 V for 10 min with a temperature gradient of 15 °C to 65 °C [36, 37]. After electrophoresis, the gel was stained with 0.05% GelRed (Biotium Inc., Fremont, CA, USA) and images were obtained using the LAS 4000 Mini (FUJIFILM, Tokyo, Japan).

### Cluster analysis

**Fig. 3:**
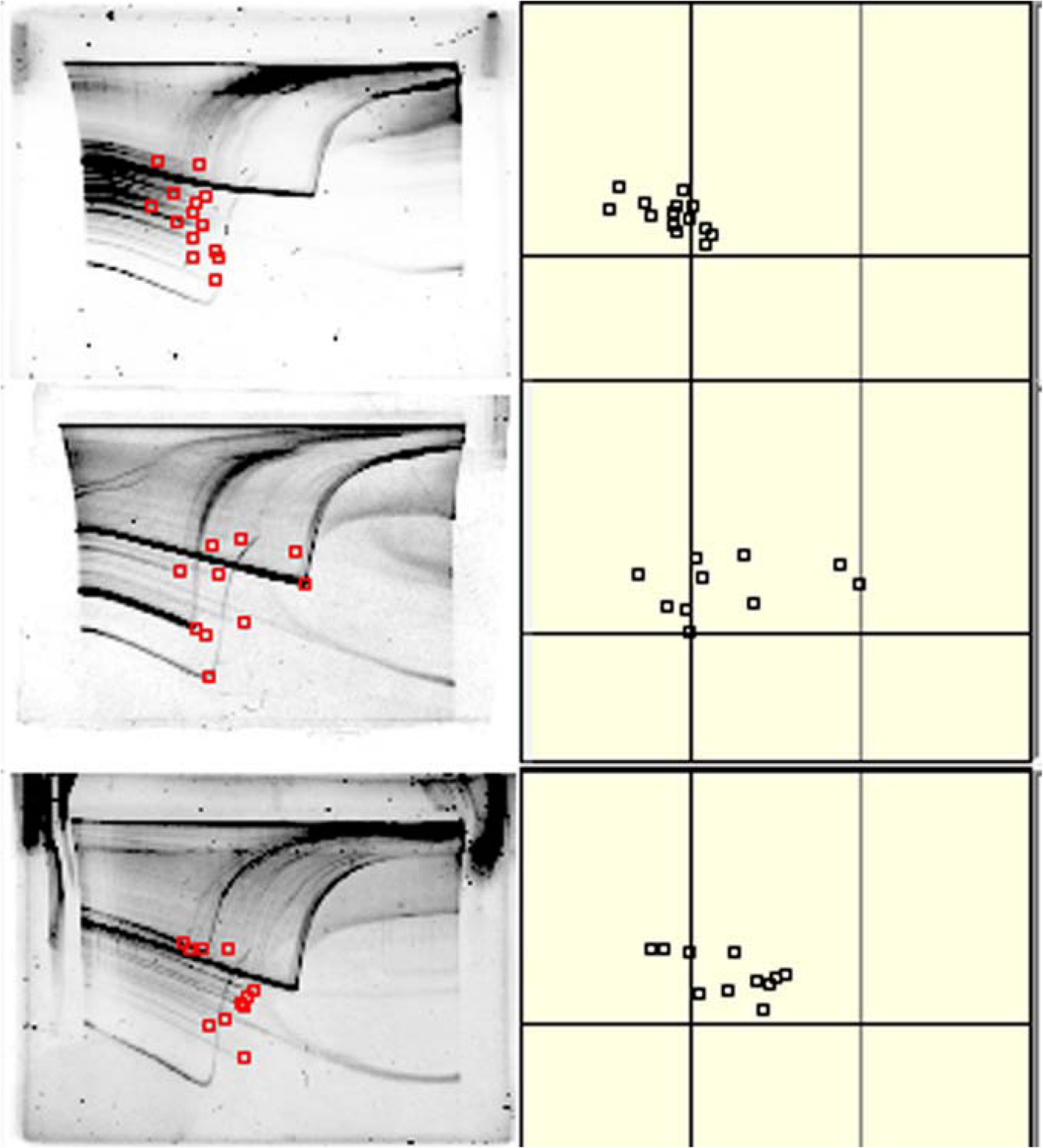
Representative images of electrophoresed gels. Human, horse mackerel, and cat sample images are shown. The electrophoresed gel image is shown on the left side, and the corrected figure is shown in the right side

From the images of the electrophoresed gels, the melting points of amplified double-stranded DNA (species identification dots: *spiddos*) were manually plotted. The *spiddos* were corrected by two reference DNA *spiddos*. Representative images of the electrophoresed gels and corrected figures are shown in Fig. 3.

The *spiddo* patterns of the samples were compared, and PaSS was calculated according to the following formula using micro-TGGE Analyzer [38–42]:

$$ \mathrm{PaSS}=1\hbox{-} \frac{1}{\mathrm{n}}\ \sum \limits_{\mathrm{i}=1}^{\mathrm{n}}\frac{\left|\overrightarrow{P_i^{(1)}}-\overrightarrow{P_i^{(2)}}\right|}{\left|\overrightarrow{P_i^{(1)}}\right|+\left|\overrightarrow{P_i^{(2)}}\right|}\kern2.759999em \left(\overrightarrow{P}=\left(\theta, \mu \right)\right) $$

PaSS takes a value of 0 to 1, where 1 indicates a perfect match. This PaSS value was analyzed using the Ward method to create a phylogenetic tree [43].

## Additional files

Additional file 1: PaSS values (SP-1). (CSV 18 kb)

Additional file 2: PaSS values (SP-2). (CSV 16 kb)

Additional file 3: PaSS values (SP-3). (CSV 18 kb)

Additional file 4: PaSS values (average of SP-1-3). (CSV 25 kb)

## Data Availability

All data generated or analyzed during this study are included in this published articles and its Additional files 1, 2, 3 and 4.

## Abbreviations

GP: Genome profiling
PaSS: Pattern similarity score
Ref1: Reference 1
Ref2: Reference 2
STR: Short tandem repeat
TGGE: Temperature gradient gel electrophoresis
